# Comparative Study on Different Modified Preparation Methods of Cellulose Nanocrystalline

**DOI:** 10.3390/polym13193417

**Published:** 2021-10-05

**Authors:** Xinhui Wang, Na Wang, Baoming Xu, Yili Wang, Jinyan Lang, Junliang Lu, Guorong Chen, Heng Zhang

**Affiliations:** 1 College of Marine Science and Biological Engineering, Qingdao University of Science & Technology, Qingdao 266042, China; wxhameq@163.com (X.W.); wlalala21@163.com (N.W.); 14763738886@163.com (B.X.); 15689122781@163.com (Y.W.); ljy17806248212@163.com (J.L.); juling_lu@163.com (J.L.); 2 Guangdong Provincial Key Lab of Green Chemical Product Technology, Guangzhou 510640, China; 3 Fujian Provincial Key Laboratory of Fire Retardant Materials, Xiamen 361005, China; grchen@xmu.edu.cn

**Keywords:** CNC, esterification reaction, graft copolymerization, hydrophobic modification

## Abstract

Different modification process routes are used to improve the modified cellulose nanocrystalline (MCNC) with higher fatty acid by esterification reaction and graft polymerization to obtain certain hydrophobic properties. Two preparation methods, product structure and surface activity, are compared and explored. Experimental results show that the modified product is still at the nanometer level and basically retains the crystal structure of the raw cellulose nanocrystalline (CNC). The energy consumption of the two preparation methods is low; however, the esterification method with co-reactant requires short reaction time, and the degree of substitution of the product is high. The modified product prepared by grafting polymerization method has a high HLB value and amphiphilicity, which can effectively reduce the surface tension of water. Therefore, it can be used as a green and environmentally friendly surface-active substance.

## 1. Preface

Nanocellulose is a degradation product of cellulose, and its basic structural unit is anhydroglucose. Nanocellulose carries a large number of hydroxyl groups. It not only inherits some of the cellulose characteristics, such as hydrophilicity, renewability, and degradability, but also has the characteristics of nanomaterials, such as high specific surface area, hyperfine structure, and high transparency. Therefore, nanocellulose is a new type of green environmental protection material with great development prospects [1,2,3].

The hydroxyl group carried by nanocellulose provides excellent modification sites. Therefore, the physical or chemical modification of nanocellulose can provide new functions and prepare new nanocellulose functional materials. Most modification techniques of cellulose are also applicable to nanocellulose given that the basic structural units of nanocellulose and cellulose are the same. Among them, physical adsorption is a commonly used polymer modification scheme. This modification method is convenient to operate, and the entire production process is simple and can effectively retain the integrity of nanocellulose. Qing et al. [4] used cetyltrimethylammonium bromide to adsorb on nanocellulose for hydrophobic drug delivery. The product has been tested, and the result proves that it has stable dispersibility and stability in organic solvents, and the raw material nanocellulose has improved in hydrophobicity. Shimizu et al. [5] made 2, 2, 6, 6-tetramethylpiperidine-1-oxy oxidized cellulose nanofibers and quaternary alkylammonium adsorbate by casting method to produce hydrophobically modified nanocellulose film. The results of hydrophobic tests show that the contact angle between the product and water has increased from the original 50° to 100°. This method improves the hydrophobicity of the product and can simply and effectively achieve the hydrophobic modification of the product. However, nanocellulose and the introduced functional groups are combined in the form of van der Waals forces or hydrogen bonds, which is instability. Because the modified products of physical modification have weak shear resistance, and the adsorbed substances are easy to dissociate under a certain external force [6].

The stability of chemically modified nanocellulose is better because the strength of chemical bonds is higher than van der Waals forces and hydrogen bonds. The commonly used chemical modification methods include oxidation, silanization, cationization, esterification, and graft copolymerization. Among them, TEMPO (2,2,6, 6-tetramethylpiperidine-1-oxygen radical) oxidation modification can oxidize the hydroxyl methyl on the surface of nanocellulose to carboxyl group and improve its water solubility. In modified polysaccharide, only the hydroxymethyl on the surface of the polysaccharide is oxidized without affecting the hydroxyl groups on the surface [7]. Araki et al. [8] used TEMPO-modified hydrochloric acid hydrolyzed nanocellulose to prepare homogeneous aqueous suspension. Silanization is widely used in surface chemical modification of cellulose whiskers, and the dispersion of the modified nanoparticles in organic solvents is improved; however, the morphology of the modified nanoparticles slightly changes, and a swelling phenomenon occurs [9]. Grunert et al. [10] modified cellulose nanocrystalline (MCNC) with trimethylsilane and compounded with cellulose acetate butyrate. The modified nanocellulose can evidently enhance the energy storage modulus of cellulose acetate butyrate. The hydroxyl groups of nanocellulose can be cationically modified by derivatization reaction. Zaman et al. [11] modified the nanocellulose hydrolyzed by sulfuric acid with glycidyl trimethylammonium chloride and adjusted the surface charge density of the modified product by changing the water content in the reaction system to be better dispersed in the water system.

Esterification and graft copolymerization are common methods for chemical modification of nanocellulose. Esterification is a chemical reaction between nanocellulose and organic acids or inorganic acids. The reaction is essentially the reaction of hydroxyl and carboxyl groups to generate ester group, sometimes with acyl halide and anhydride as the reactants; however, the final result is the generation of ester group. Lakovaara et al. [12] obtained high strength and sustainable films with increased hydrophobicity and good mechanical properties by the esterification of cellulose nanofiber and all-cellulose composite films with n-octylsuccinic anhydride. In addition, the experimental method is relatively mild. Beaumont et al. [13] presented a general concept of wet surface esterification of cellulose using acyl imidazoles, which means acetyl groups are introduced directly onto never-dried, water-swollen cellulose fibres. This method can obtain cellulose fibers with higher hydrophobicity in simple and gentle process. Shang et al. [14] prepared super-hydrophobicity/super-oleophilicity with good adsorption capacity through the freeze-drying process and subsequent esterification. The reaction process of it was facile and environmental-friendly approach. Spinella et al. [15] prepared functionalized modified product by direct melt blending of nanocellulose and lactic acid through Fischer esterification reaction, which improved the dispersion of original nanocellulose in polymer matrix, increased the contact angle with water, and expanded the application range of this material. Yuan et al. [16] acetylated nanocellulose by using alkyl succinic anhydride. After modification, the hydrophobicity of nanoparticles was significantly improved and was easily dispersed into polar solvents with different dielectric constants. Menezes et al. [17] reacted the nanocellulose with organic fatty acid chlorides of different hydrocarbon chain lengths to prepare esterification-modified products with high substitution degree and high graft density. Berlioz et al. [18] reacted BNC with hexadecyl chloride vapor to obtain the same esterified product of nanocellulose with high substitution degree. This reaction is a highly efficient solvent-free esterification reaction, and the crystal structure of the modified nanocellulose is insignificantly changed. Eyholzer et al. [19] used sulfuric acid as the catalyst to transfer the reaction of ester group of nanocellulose in hexane–alcohol medium, and hexyl monomer replaced carboxymethyl through carboxymethylation.

Graft copolymerization can directly react the functional groups of the graft with the active hydroxyl group of the nanocellulose to form the modified products, or it can initially react with the active hydroxyl group of the nanocellulose by the small molecular monomer, and then perform chemical modification by the polymerization of the small molecular monomer grafted on the nanocellulose, namely, chain growth. Goffin et al. [20] grafted PLA on the surface of cellulose nanocrystalline through ring-opening polymerization. The length of grafting chain in the modified products is short due to the crystallization of PLA on the surface of nanocellulose. Westlund et al. [21] prepared graft products with special functions by modifying nanocellulose with tris-[2-(dimethylamino) ethyl] amine as ligand and 11-(4′-cyanophenyl-4″-phenoxy) undecyl acrylonitrile and methyl acrylate as monomers. Lee et al. [22] grafted acrylamide onto the surface of nanocellulose by ultraviolet light initiation by using benzophenone as initiator. The grafting ratio of modified products increased with the increase in initiator concentration. The introduction of acrylamide chain improved the dispersion ability of nanocellulose. Morandi et al. [23] grafted polyphenylene onto the surface of nanocellulose by using ethyl bromoisobutyrate as initiator. The modified products showed chiral nematic structure in thermotropic and lyotropic liquid crystal and had strong adsorption for 1,2, 4-trichlorobenzene.

Among nanocellulose modification methods, esterification and graft copolymerization have more mature reaction routes and do not require harsh reaction conditions; thus, they have the most industrial application prospects. In our previous studies, we investigated the graft copolymerization method of nanocellulose, grafting high fatty acids with FeSO_4_/H_2_O_2_ system or 2(NH_4_)_2_SO_4_·Ce(SO_4_)_2_·4H_2_O as initiator. Under the same reaction conditions, the initiation efficiency of FeSO_4_/H_2_O_2_ was better than that of tetravalent cerium ion in ammonium cerium sulfate, and the degree of substitution (DS) of the obtained product was relatively high [24,25,26]. However, only a few studies compared the esterification method with that using co-reactant. The esterification reaction modified cellulose nanocrystalline has the characteristics of facile and eco-friendly. Therefore, low reaction temperature and green reagent were used in this experiment. Nanocellulose and higher fatty acids were used as raw materials to graft alkanes onto nanocellulose by introducing co-reactants and initiators, respectively. The performance of reaction products was evaluated by comparing the degree of difficulty of the two reaction types. The differences in surface activity of the products were analyzed on the basis of the reaction mechanism, thereby providing a feasible process route for the modification of nanocellulose.

## 2. Preparation and Characterization of Cellulose Nanocrystalline (CNC) and Modified Products

### 2.1. Chemicals and Materials

Trifluoroacetic anhydride was purchased from Shanghai Aladdin Biochemical Technology Co., Ltd. Ferrous sulfate was obtained from China Shanghai Pierce Chemical Reagent Co., Ltd. Hydrogen peroxide at 30% (*w*/*w*) was purchased from Tianjin Dingshengxin Chemical Co., Ltd. Chloroform, sodium hydroxide, and absolute ethanol were purchased from Sinopharm Chemical Reagent Co., Ltd. Lauric acid was obtained from Tianjin BASF Chemical Co., Ltd. Palmitic acid was obtained from Tianjin Bodi Chemical Co., Ltd. Stearic acid was purchased from Tianjin Beichen Fangzheng Reagent Factory. Concentrated sulfuric acid, hydrochloric acid, was purchased from Yantai Sanhe Chemical Reagent Co., Ltd. These chemicals are all analytical pure. Pharmaceutical grade microcrystalline cellulose was purchased from Chengdu Kelong Chemical Reagent Factory. The MD3500 dialysis bag was purchased from an American company, Viskase.

### 2.2. Preparation of CNC

Concentrated sulfuric acid aqueous solution at 60% (*w*/*w*) was prepared and naturally cooled. It was directly added to a flask containing microcrystalline cellulose (MCC) when its temperature dropped to approximately 45 °C. The magnetic heater was heated to 45 °C, and the speed was adjusted to 500 rpm. Timing after the mixture is evenly stirred, the whole reaction is protected by nitrogen. After 2.5 h, heating was stopped, the mixed solution was transferred to a beaker, and a large amount of deionized water was added to terminate the acid-catalyzed hydrolysis reaction. Finally, the milky white product mixed with microcrystalline cellulose and CNC obtained after adding deionized water was transferred to a 50 mL centrifuge tube. The turbid liquid was centrifuged at the speed of 5000 rpm to separate the upper layer of acid-containing waste liquid, and deionized water was added to continuously dilute the concentration of the sulfuric acid aqueous solution. This process is repeated until the supernatant liquid became turbid, and then the supernatant liquid containing the desired CNC was collected.

The collected upper layer of CNC colloid was concentrated by vacuum distillation, and then transferred to the dialysis bag (molecular weight 3500) under 30 °C deionized water environment with magnetic stirring; dialysis was repeated until the pH became neutral. Finally, a transparent and light blue glowing CNC colloid was obtained.

### 2.3. Preparation of MCNC

#### 2.3.1. Preparation of MCNC by Esterification Reaction

Trifluoroacetic anhydride was used as the co-reactant to activate three higher fatty acids in situ to produce a mixed acid anhydride intermediate. Then, the dried CNC powder was added to a four-necked flask containing mixed acid anhydride intermediate to generate MCNC at different temperatures and reaction times. The system should be anhydrous and ethanol-free because trifluoroacetic anhydride is chemically active and can react with water and absolute ethanol. Through preliminary experiments and exploration, chloroform was selected as the liquid phase system of the reaction. The entire reaction process was under nitrogen protection. When the reaction was over, the mixture was cooled to room temperature, and the substances that did not participate in the reaction were removed by alternate washing with absolute ethanol and water, and the resulting product was separated. Then, ethanol was used as the solvent to extract the product using a Soxhlet extractor. Finally, the product was vacuum dried at 40 °C. The reaction mechanism is shown in Equation (1).
(1)
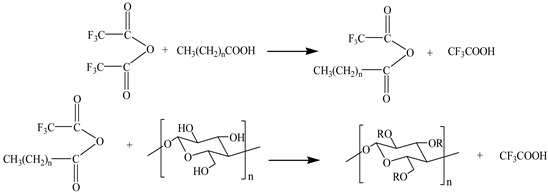


#### 2.3.2. Preparation of MCNC by Graft Copolymerization

An appropriate amount of self-made CNC colloidal solution was obtained and mixed with an ethanol solution mixed with dissolved fatty acids. The reaction was carried out in a flask, stirring and mixing continuously for 0.5 h in the water bath environment at 50 °C to obtain even mixture. Then, a quantitative initiator was added, and the reaction temperature and time were changed to carry out the chemical modification reaction of CNC. The entire reaction process was under nitrogen protection. After the reaction, a mixed suspension of MCNC was obtained, and the suspension was separated and washed alternately with alcohol and water. Finally, the product was vacuum dried at a temperature of 40 °C.

In the process of MCNC preparation with FeSO_4_/H_2_O_2_ as the initiator, Fe^2+^ and H_2_O_2_ undergo a redox reaction, thereby triggering H_2_O_2_ to generate active free radicals. The free radicals cause fatty acids and CNC to form two active free radicals. Finally, these active free radicals react with each other to complete the chemical modification of CNC [27,28,29]. The reaction mechanism of this process is shown in Equation (2).
(2)
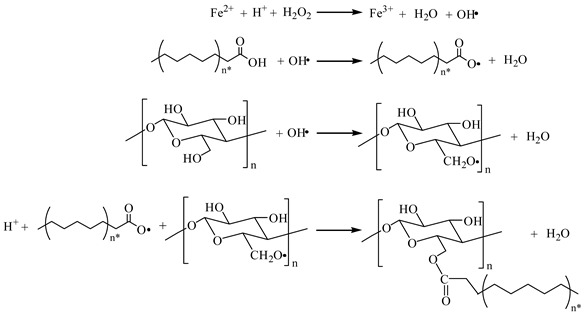


### 2.4. Test Method

#### 2.4.1. Determination and Calculation of DS of MCNC

The synthesized MCNC was repeatedly washed with ethanol and deionized water, filtered, and dried to obtain the product, and the DS was determined by the saponification method [30,31].

#### 2.4.2. X-ray Diffraction

CNC and MCNC powder samples were obtained for X-ray diffraction measurement. At room temperature, the measured substance was tested with a DX-2700 XRD tester (Bruker Corporation, Ettlingen, Germany) at a scanning rate of 0.02°/min. The diffraction setting parameters are as follows: Cu target, 40 kV, and 30 mA; the diffraction angle 2θ range is set to 10°–60°.

The crystallinity index CrI value can be calculated by Segal equation (Equation (3)) [32], as follows:

(3) $$\mathrm{CrI}=\frac{I_{002}{-I}_{\mathrm{am}}}{I_{002}}\times 100\%$$

(CrI: relative crystallinity index; I_002_: maximum intensity of 002 lattice diffraction; I_am_: diffraction intensity of the same unit at 2θ = 18°)

#### 2.4.3. Determination of Particle Size

CNC colloid and MCNC suspension are diluted and ultrasonically dispersed for testing. Malvern Zetasizer Nano-ZS90-type Malvern laser particle size analyzer (Malvern (China) instrument Co. Ltd., Shanghai, China) is used. The particle refractive index is set to 1.470, water is used as the dispersant, the temperature of the test system is 25 °C, the count rate is 349.8 kcps, and the time is set to 60 s.

#### 2.4.4. Calculation of the HLB Value of MCNC

Equation (4) is used to calculate the HLB value of the MCNC.

(4) $$HLB=\frac{\mathrm{Hydrophilic}\;\mathrm{group}\;\mathrm{quality}}{\mathrm{Surfactant}\;\mathrm{quality}}\times \frac{100}{5}\;=\frac{\mathrm{The}\;\mathrm{quality}\;\mathrm{of}\;\mathrm{the}\;\mathrm{hydrophilic}\;\mathrm{group}}{\mathrm{Lipophilic}\;\mathrm{base}\;\mathrm{quality}+\mathrm{Hydrophilic}\;\mathrm{group}\;\mathrm{quality}}\times \frac{100}{5}\;=\frac{A-DS}{\left(B-1\right)\times DS+A}\times \frac{100}{5}$$

(A: relative molecular mass of glucoside unit; B: relative molecular mass of fatty acid groups (C_12_H_23_O, C_16_H_33_O, C_18_H_37_O) connected to CNC in the form of ester bonds; DS: degree of substitution of MCNC).

#### 2.4.5. Determination of Surface Tension

The CNC and MCNC test liquids with different contents are configured, and the surface tension of the QBZY-2 automatic surface tension meter (Shanghai Fangrui Instrument Co., Ltd., Shanghai, China) was used to determine the surface tension.

## 3. Results and Discussion

### 3.1. Factors Affecting the DS of MCNC

The saponification method was used to determine the DS to explore the influence of different reaction conditions on the DS of the modified product. The co-reactant or initiator, three higher fatty acids, and CNC are combined, and the corresponding temperature change interval and time change interval are selected to prepare MCNC under different reaction conditions.

#### 3.1.1. Effect of Co-Reactant Content on the DS of MCNC

Trifluoroacetic anhydride acts as a co-reactant to react with three types of higher fatty acids to generate active intermediates, namely, mixed acid anhydrides. MCNC was prepared by the reaction of mixed anhydride and CNC. The amount of fatty acid was the same as that of the co-reactant according to the esterification reaction mechanism. When lauric acid was used as the grafting substance, the temperature should be controlled at 50 °C, and the reaction time should be 5 h. When palmitic acid and stearic acid were used as grafting substances, the reaction temperature was controlled at 55 °C and the reaction time was 6 h. The test results are as follows.

**Figure 1:**
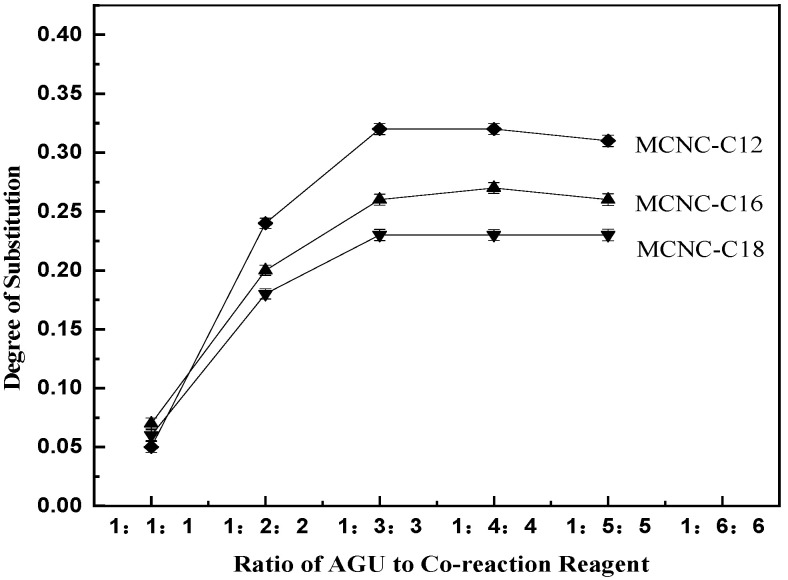
Effect of the ratio of co-reaction reagent on substitution degree of MCNC. (MCNC-C12: MCNC grafted with lauric acid; MCNC-C16: MCNC grafted with palmitic acid; MCNC-C18: MCNC grafted with stearic acid.)

Figure 1 shows that the DS of MCNC is related to the feed ratio of the substances participating in the reaction and the hydrocarbon chain length of the hydrophobic groups of MCNC. As the adding ratio of anhydroglucose unit (AGU), co-reactant and fatty acid increases, the DS of MCNC gradually increases. When the feeding ratio reaches 1:3:3, the curve tends to be flat. On the basis of the esterification reaction mechanism, the increase in the feed ratio increases the concentration of the mixed acid anhydride in the reaction system, thereby increasing the probability of reacting with the CNC hydroxyl group. Thus, the degree of product substitution increases. However, when the feeding ratio is excessive, although the concentration of the mixed acid anhydride still increases on a macro scale, the contact concentration of the mixed acid anhydride with the hydroxyl group gradually becomes saturated; thus, the DS does not change evidently. The DS of MCNC-C12 is higher than that of MCNC-C16 and MCNC-C18; it is determined by the steric hindrance of higher saturated fatty acids. With the continuous increase in fatty acid hydrocarbon chain length, its steric hindrance effect gradually becomes evident, inhibiting the increase in substitution degree. Therefore, the DS of the three MCNC from high to low is MCNC-C12, MCNC-C16, and MCNC-C18, under the same conditions.

#### 3.1.2. Effect of Initiator and Content on the DS of MCNC

FeSO_4_/H_2_O_2_ was selected as the initiator, the content of the added initiator was changed at a specific reaction temperature and time, and lauric acid, palmitic acid, and stearic acid were used to react with the CNC. CNC reacted with lauric acid in the 50 °C water bath environment for 8 h, CNC and palmitic acid reacted in the 55 °C water bath environment for 20 h, and CNC reacted with stearic acid in the 60 °C water bath environment for 24 h. Different initiator types were added, and the addition amount was changed to prepare MCNC with different DS under these reaction conditions. The DS is measured, and the results are shown in the figure.

**Figure 2:**
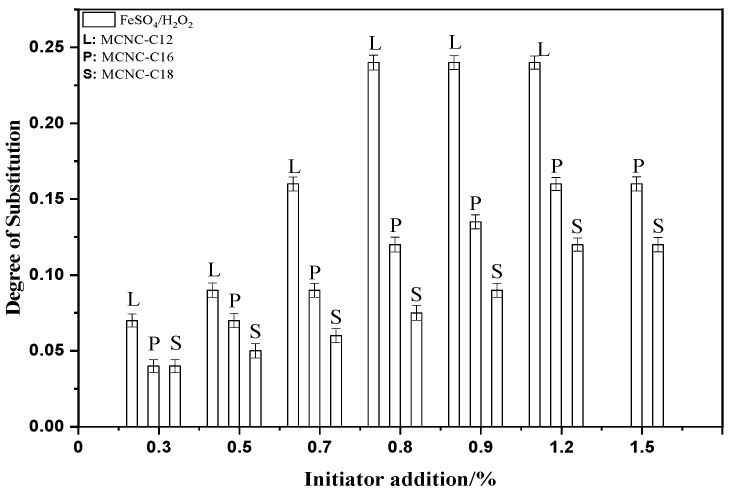
Effect of initiator type and dosage on substitution degree of MCNC. (MCNC-C12: MCNC grafted with lauric acid; MCNC-C16: MCNC grafted with palmitic acid; MCNC-C18: MCNC grafted with stearic acid.)

Figure 2 shows that under the FeSO_4_/H_2_O_2_ redox initiation system, as the amount of initiator increases, the DS of lauric acid, palmitic acid, and stearic acid esterified CNC initially increases, and then gradually tends to be flat. When the amount of initiator is 0.8%, lauric acid as the graft can obtain the best DS of 0.24 under this preparation method. In addition, the amount of initiator continues to increase, and the DS tends to be stable. The substitution effect of palmitic acid and stearic acid is the same as that of lauric acid. The best DS is obtained when the dosage of the initiator is 1.2%. The substitution degrees of palmitic acid and stearic acid are 0.16 and 0.12, respectively.

The main reason for this phenomenon is the steric hindrance of the system on the glucoside unit. When the amount of initiator is not optimal, the initiation efficiency of the reaction system gradually increases as the content of initiator increases, the concentration of initiated active free radicals increases, and the degree of reaction substitution increases. In addition, the FeSO_4_/H_2_O_2_ system generates free radicals on the primary hydroxyl group at the C_6_ position of the glucoside unit. When the amount of initiator is higher than the optimal amount, the DS of the product tends to be stable. At this time, although increasing the amount of initiator can still increase the concentration of free radicals, the space steric hindrance effect of higher fatty acids greatly affects the esterification efficiency between free radicals, and the steric hindrance effect increases with the increase in the length of the fatty acid hydrocarbon chain, thereby limiting the continued increase in the DS of the product.

Therefore, the steric hindrance effect of higher fatty acid becomes an important factor affecting the DS when the free radical content reaches a certain concentration. Under the FeSO_4_/H_2_O_2_ system, the DS of lauric acid, palmitic acid, and stearic acid MCNC also decreases.

#### 3.1.3. Effect of Reaction Time on Substitution Degree of MCNC

Lauric acid was used as raw material, and trifluoroacetic anhydride was used as co-reaction agent; they are mixed in a molar ratio of 1:1. The mixture was prepared at 4 h, 5 h, 6 h, 7 h, and 8 h, by changing the reaction time in a water bath at 50 °C with the molar ratio of 3:1 to CNC. The preparation method of palmitic acid and stearic acid as raw materials was similar to that of lauric acid, but the reaction temperature was raised to 55 °C. After the reaction, an appropriate amount of stearic acid MCNC was obtained, and the DS was tested by saponification method.

With 0.8% FeSO_4_/H_2_O_2_ as the initiator, lauric acid was reacted with CNC in a water bath at 50 °C to prepare MCNC-C12. The reaction time was set at 6 h, 7 h, 8 h, 9 h, and 10 h, according to the same experimental method to prepare MCNC-C16 and MCNC-C18. The reaction temperature was set to 55 °C, and 1.2% FeSO_4_/H_2_O_2_ was selected as the initiator. The reaction times of 16 h, 18 h, 20 h, 22 h, and 24 h were set for the graft copolymerization of CNC with palmitic acid as the raw material. The reaction temperature was set to 55 °C, and 1.2% FeSO_4_/H_2_O_2_ was selected as the initiator. The reaction times of 16 h, 18 h, 20 h, 22 h, 24 h, and 25 h were set for the graft copolymerization of CNC with stearic acid as the raw material. After the reaction, an appropriate amount of stearic acid MCNC was obtained, and the DS was tested by saponification method. The test results are as follows.

**Figure 3:**
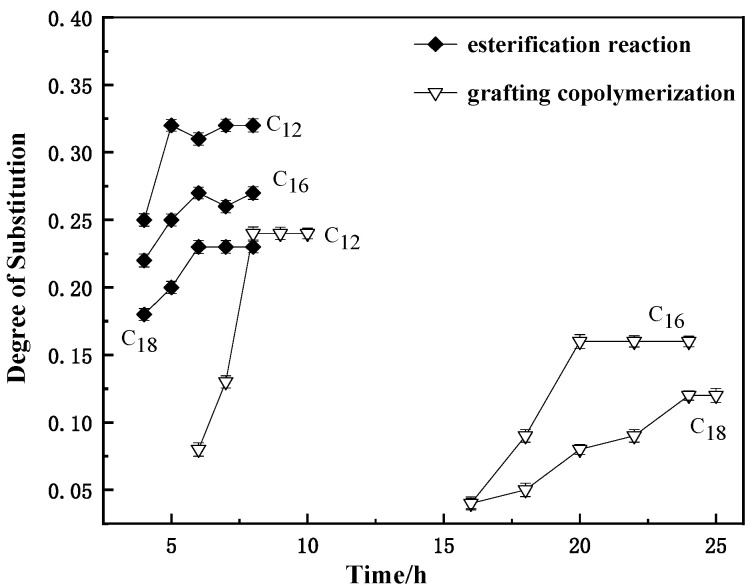
Effect of reaction time on substitution degree of MCNC.

Figure 3 shows that under the same conditions, the DS of lauric acid was higher than that of palmitic acid, whereas that of stearic acid was the lowest. The esterification produced the mixture of anhydrides with higher activity than fatty acids in the first place; thus, its substitution degree is higher than the graft copolymerization under the same conditions. Moreover, the time interval for the preparation of three fatty acid esterified CNC by this method is more concentrated. However, in the graft copolymerization, the reaction time was longer because the steric hindrance caused by the over-long hydrocarbon chains of palmitic acid and stearic acid was much higher than that of lauric acid. In addition, their reaction activity was lower than that of anhydride. In general, the reaction time of the modified products of lauric acid was lower than that of the modified products of the two other acids. In summary, in the system with trifluoroacetic anhydride as co-reactive agent, the optimal reaction time with lauric acid as the raw material is 5 h, palmitic acid as the raw material is 6 h, and stearic acid as the raw material is 6 h. However, in the initiator system, the optimal reaction time of the three fatty acids was 8 h, 20 h, and 24 h.

#### 3.1.4. Effect of Temperature on the DS of MCNC

Trifluoroacetic anhydride was considered the co-reactant, and CNC was mixed at a molar ratio of 3:1. The amounts of fatty acids involved in the reaction were the same as that of the co-reactant. When lauric acid was used, the reaction time was controlled at 5 h, and the reaction temperatures were set at 40 °C, 45 °C, 50 °C, 55 °C, and 60 °C. When soft fatty acid was used, the reaction time was controlled at 6 h, and the reaction temperatures were set at 40 °C, 45 °C, 50 °C, 55 °C, and 60 °C. When stearic acid was used, the reaction time was controlled at 6 h, and the reaction temperatures were set at 40 °C, 45 °C, 50 °C, 55 °C, and 60 °C; when stearic acid was used, the reaction time was controlled at 6 h, and the reaction temperatures were set at 40 °C, 45 °C, 50 °C, 55 °C, and 60 °C.

At 8 h reaction time, CNC was graft-modified with lauric acid by adding 0.8% FeSO_4_/H_2_O_2_ at 35 °C, 40 °C, 45 °C, 50 °C, 55 °C, 60 °C, and 65 °C. At 20 h reaction time, CNC was graft-modified with soft stearic acid by adding 1.2% FeSO_4_/H_2_O_2_ at 35 °C, 40 °C, 45 °C, 50 °C, 55 °C, 60 °C, and 65 °C. At 24 h reaction time, CNC was graft modified with stearic acid by adding 1.2% FeSO_4_/H_2_O_2_ at 35 °C, 40 °C, 45 °C, 50 °C, 55 °C, 60 °C, and 65 °C. Appropriate amounts of the prepared fatty acid modification products were taken and tested for their DS by saponification method.

**Figure 4:**
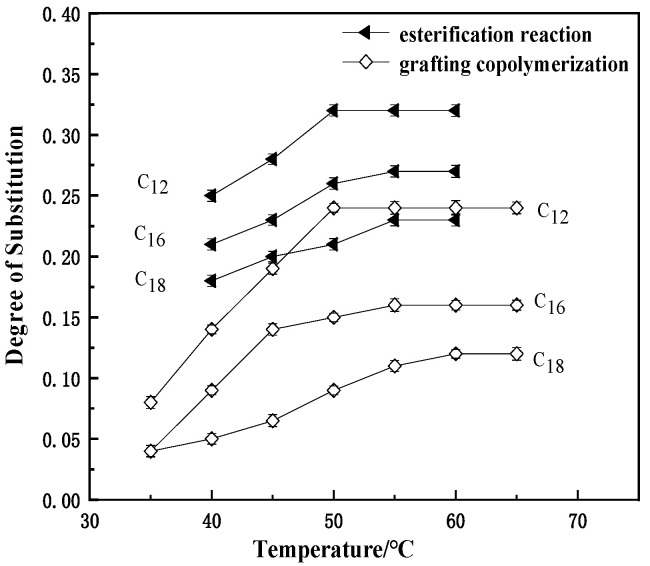
Effect of reaction temperature on substitution degree of MCNC.

Figure 4 reflects the effect of reaction temperature on the degree of product substitution when grafting CNC by both preparation methods. The trend of the curves in the figure is the same as that of the effect of reaction time on the DS of the modified products. Lauric acid has a higher DS than soft and stearic acid under the same conditions due to the steric hindrance effect of the polymer. Relatively high DS were obtained at 50 °C to 60 °C for both preparations, but the reaction temperature was relatively higher for stearic acid because as the hydrocarbon chains of the fatty acids involved in the reaction grow, their steric hindrance becomes progressively greater. When the molecular weight of the fatty acid reaches 18 carbons, the steric hindrance becomes an important factor in the DS of the modified reaction, and the reaction system requires a higher temperature to better activate the reactants.

In summary, when using trifluoroacetic anhydride as the co-reactant, the optimum reaction conditions for MCNC-C12 were 50 °C, 5 h, and 1:3 feeding ratio, and the degree of product substitution was 0.32. The optimum reaction conditions for MCNC-C16 were 55 °C, 6 h, and 1:3 feeding ratios; the DS was 0.27. The optimum reaction conditions for MCNC-C18 were 55 °C, 6 h, and 1:3 feeding ratio, and the degree of product substitution was 0.23. In the FeSO_4_/H_2_O_2_ redox system, the highest DS of 0.24 was achieved by adding 0.8% initiator at a reaction temperature of 50 °C for 8 h. The highest DS of 0.24 was achieved by adding 1.2% initiator at a reaction temperature of 55 °C for MCNC-C16 for 20 h. In the FeSO_4_/H_2_O_2_ redox system, the highest DS of 0.24 was achieved by adding 0.8% initiator at a reaction temperature of 50 °C for MCNC-C12. The maximum DS was 0.16 for 20 h at 55 °C with 1.2% initiator and 0.12 for 24 h at 60 °C with 1.20% initiator for MCNC-C18. The differences in the DS between the two methods were due to the different principles of modifying CNC with co-reactants and initiators. The nature of the modified product by introducing a co-reactant is the reaction between the mixed anhydride and the hydroxyl groups of the CNC, which are at the C_6_, C_2_, and C_3_ positions. On the contrary, the use of an initiator for the preparation of the modified product involves the initiation of the hydroxyl group at the C_6_ position, and the number of reaction sites is different between the two methods; hence a difference is found in the DS.

### 3.2. X-ray Diffraction Analysis of MCNC

**Figure 5:**
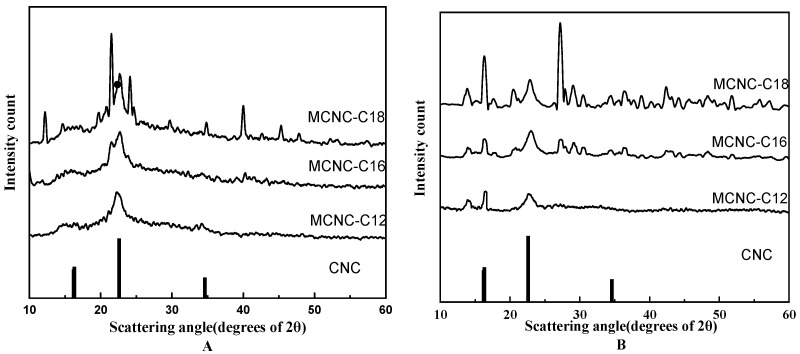
X-ray diffraction pattern of fatty acid MCNC. ((**A**): diffraction curve of MCNC crystal prepared by esterification method, and (**B**): diffraction curve of MCNC crystal prepared by graft copolymerization method).

X-ray diffraction analysis of CNC and MCNC prepared by different methods. The test results are shown in the following Figure 5:

Regardless of whether the esterification substance with CNC is lauric acid, palmitic acid, or stearic acid, the essence of the grafted CNC is the same. The difference in steric hindrance caused by the chain length causes different crystallinity. Therefore, the XRD diffraction peak value of various products prepared under the same system is different, but the peak position is approximately the same. The results show that the diffraction peak of the MCNC is weakened at the intensity of I_002_, and the crystallinity of the modified products decreases. However, the products of the grafted MCNC with three types of advanced fatty acids still have diffraction peaks at the intensity of I_002_, I_am_, and I_004_, indicating that the products still maintain the basic crystal structure of CNC. The new diffraction peak generated by MCNC and the decrease in the area of the diffraction peak at the intensity of I_002_ also prove the chemical modification of CNC.

### 3.3. MCNC Particle Size Test

**Figure 6:**
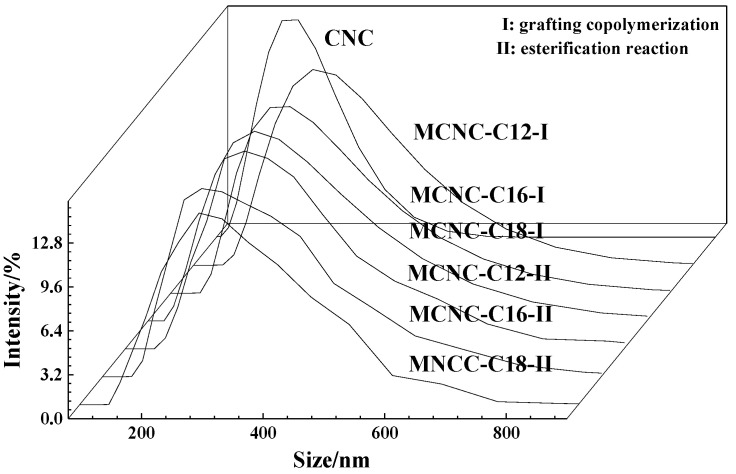
Particle size distribution of MCNC.

MCNC was prepared under the optimal reaction conditions to detect the particle size of CNC and three types of MCNC, using trifluoroacetic anhydride as co-reactant and FeSO_4_/H_2_O_2_ as initiator. The test results are as follows:

Figure 6 shows that the average particle size of CNC modified by fatty acids exceeds the average particle size of the original CNC (168 nm), which is in the range of 240–260 nm. However, the specific surface area is lower than the value of unmodified CNC (19,260 m^2^/kg), which is in the range of 17,400–17,500. In general, regardless of the advanced fatty acid and method selected to prepare MCNC, the difference between the particle size, particle size distribution coefficient, and specific surface area is very small, and can even be approximately considered the same. However, because the long chain of carbon and hydrogen replaced the original hydrogen atoms after esterification of fatty acids and CNC, the molecular weight increased to varying degrees, and the spatial scale of molecular structure increased. Thus, the particle size of the modified products still increased slightly; it was higher than that of the unmodified CNC. Nevertheless, the esterified cellulose particles are still nanometer in size. The figure shows that the grafting of long-chain fatty acids did not significantly increase the original nanometer particle size, but only increased the particle size in a small range. In addition, modified products with wider particle size distribution are greater than the original CNC size distribution coefficient. This finding shows that during the esterification reaction of CNC, a certain degree of flocculation has occurred. The reduction of the specific surface area of the MCNC causes the original CNC to be grafted with long hydrocarbon chains, weakening their reactivity to a certain extent. Therefore, CNC is still at the nanoscale level and has large specific surface area after esterification and grafting of carbon and hydrogen long chains. This condition lays a good foundation for their subsequent functional applications.

### 3.4. Calculation of HLB Value of MCNC

In accordance with the HLB value equation (Equation (4)), the HLB values of MCNC under various optimal reaction conditions were calculated and shown in Table 1.

By comparison, the HLB values of MCNC prepared by grafting copolymerization are slightly higher than that of MCNC prepared by esterification. The HLB values of MCNC are affected by the DS and the length of the grafted hydrocarbon chain. With the increase in DS, the HLB values show a decreasing trend. The increase in DS increases the number of hydrophobic groups, and the modified products become more lipophilic. Thus, the MCNC gradually tend to be hydrophobic, and the HLB values decreases. However, due to the different molecular weights of the grafted fatty acids, the calculation results of HLB value would be affected to some extent. In addition, the phenomenon indicates that the HLB value of low-DS MCNC is higher than that of low-DS MCNC. In general, all modified products are hydrophilic and lipophilic. The raw CNC contains a large number of unsubstituted hydroxyl groups due to the low DS of MCNC. Thus, modified products tend to be hydrophilic. Therefore, the amphiphilicity of MCNC is determined by the DS and the length of the grafted hydrocarbon chain.

### 3.5. Influence of MCNC Concentration on Surface Tension of Water

CNC and MCNC were configured into different concentrations of suspension to be tested. During the test, the suspension was pretreated with ultrasound, and the surface tension of suspension was measured at 25 °C. The following results were obtained.

**Figure 7:**
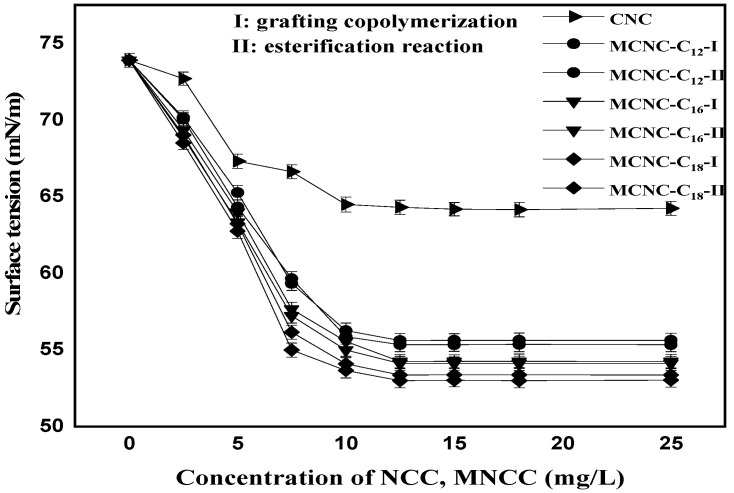
Effect of MCNC concentration on surface tension of MCNC suspension.

Figure 7 shows that CNC and their modified products can effectively reduce the surface tension of water, and the modified products have a more evident reduction effect on the surface tension. With the gradual increase in the concentration of various substances in water, the decreasing trend of surface tension changes from rapid to smooth. When a certain concentration is reached, continuously increasing its concentration does not cause a significant change in the surface tension of the water. The concentration value at this point is called the critical micelle concentration (CMC). In Figure 7, the CMC of the CNC and the MCNC appear near 12.5 mg/L, and the surface tension reduction intensity increases as the length of the hydrocarbon chain grafted on the CNC increases. Thus, MCNC-C18 has the best effect on reducing the surface tension of water, followed by MCNC-C16 and MCNC-C12. The figure shows that the carbon chain length slightly affects the critical micelle concentration because the main molecular chain skeleton of CNC is a macromolecular structure. At the same concentration of the same modified products, the esterification method has better surface tension reduction effect than the grafting copolymerization method. Its essence is due to the higher DS of the products prepared by the esterification, and the change in the carbon chain length slightly affects the overall structure. In addition, the structure analysis indicates that the MCNC cannot ionize in water; thus, MCNC belongs to the non-ionic surfactant.

## 4. Conclusions

The MCNC prepared by esterification and grafting copolymerization is compared. Both methods can complete the reaction with low energy consumption, but the preparation time of the esterification method is shorter and the DS of MCNC is higher. The X-ray diffraction and particle size analysis of the modified products show that the products prepared by the two methods maintain the crystal structure of the raw CNC, and they are at the nanometer level. The measurement results of HLB value and surface tension prove that the modified products have hydrophilic and lipophilic properties, and the effect of reducing the surface tension of water is better than that of raw CNC. Because of good biodegradability, the modified product can be used as a new type of green surface-active substance, which are endowed with emulsion, dispersion, wetting, washing, sterilization, water resistant, antistatic, solubilization and stable features. Further, it will be widely concerned in food, chemical, agriculture, construction and other fields, to achieve high value utilization of biomass resources.

## Figures and Tables

**Table 1:** HLB value of fatty acid graft MCNC.

MCNC	HLB Value	
	Esterification	Grafting Copolymerization
MCNC-C12	14.68	15.73
MCNC-C16	14.29	16.17
MCNC-C18	14.49	16.69

## Data Availability

The data presented in this study are available on request from the corresponding author.
